# SPOCK2 Serves as a Potential Prognostic Marker and Correlates With Immune Infiltration in Lung Adenocarcinoma

**DOI:** 10.3389/fgene.2020.588499

**Published:** 2020-11-10

**Authors:** Jinming Zhao, Ming Cheng, Junda Gai, Ruochen Zhang, Tengjiao Du, Qingchang Li

**Affiliations:** ^1^Department of Pathology, College of Basic Medical Sciences, China Medical University, Shenyang, China; ^2^Department of Pathology, The First Affiliated Hospital of China Medical University, Shenyang, China; ^3^Department of Chronic Disease Epidemiology, Yale School of Public Health, Yale University, New Haven, CT, United States

**Keywords:** SPOCK2, tumor-infiltrating immune cell, prognosis, lung adenocarcinoma, bioinformatics

## Abstract

Lung adenocarcinoma (LUAD) is one of the major types of lung cancer. Tumor-infiltrating immune cells (TIICs) are positively associated with overall survival (OS) in LUAD. The SPARC/osteonectin, cwcv and kazal-like domains proteoglycan 2 (SPOCK2) is a complex type of secreted proteoglycan involved in forming a protective barrier against viral infection. The purpose of this study was to investigate the relationship between SPOCK2 and TIICs and the prognostic role of SPOCK2 in LUAD. The GEPIA2, GEO, CPTAC, and HPA databases were analyzed to examine both the mRNA and protein expression of SPOCK2 in LUAD. GEPIA2 and the Kaplan-Meier Plotter (KM Plotter) were used to evaluate the prognostic value of SPOCK2 in LUAD patients. TCGA data were examined for a correlation between SPOCK2 expression and clinical characteristics. Gene enrichment analyses were performed to explore the underlying mechanism of SPOCK2 based on LinkedOmics. RegNetwork was used to predict the regulators of SPOCK2. The correlation between SPOCK2 and TIICs, including immune infiltration level and relative proportion was investigated via TIMER. KM Plotter was also used to evaluate the prognostic role of SPOCK2 expression in LUAD with enriched and decreased TIIC subgroups. We found SPOCK2 was significantly downregulated in LUAD compared with that in non-tumor controls and was correlated with clinical parameters. Moreover, a high SPOCK2 expression level predicted better survival. The SPOCK2-associated regulatory network was constructed. SPOCK2 influenced the TIIC infiltration level and relative proportion in LUAD. Furthermore, a high SPOCK2 expression level was associated with a favorable prognosis in enriched CD4 + T cells and macrophage subgroups in LUAD. In conclusion, a high level of SPOCK2 expression predicted favorable prognosis and was significantly correlated with TIICs in LUAD. Therefore, the expression of SPOCK2 may affect the prognosis of LUAD partly due to TIICs.

## Introduction

Lung cancer has become the most common cancer type and causes the largest number of cancer-related deaths in the world (Siegel et al., 2019). Lung adenocarcinoma (LUAD) is a crucial histological phenotype of lung cancer (Testa et al., 2018). Immunotherapy is a promising treatment strategy for LUAD, and clinical trials of immunotherapy are underway. The effect of immunotherapy on LUAD progression and outcome depends on both the cancer phenotype and tumor-infiltrating immune cell (TIIC) subsets in the tumor microenvironment. It was reported that TIICs are positively associated with better survival in LUAD, which highlights the importance of TIICs in the clinical outcomes of LUAD patients (Vafadar, 2019).

SPARC (osteonectin), cwcv and kazal-like domains proteoglycan 2 (SPOCK2) is known as a secreted protein that is acidic and cysteine-rich, playing a significant role in the development and progression of ovarian cancer, endometrial cancer, and prostate cancer (Liu et al., 2019; Lou et al., 2019; Ren et al., 2019). It was reported that SPOCK2 can prevent viral infection in lung epithelial cells (Ahn et al., 2019). However, its association with prognosis in LUAD and its possible immune mechanisms are still elusive. In this study, we aimed to examine these immune mechanisms and the prognostic role of SPOCK2 in LUAD.

We first analyzed the differential mRNA expression of SPOCK2 between LUAD and normal lung tissues in the Gene Expression Profiling Interactive Analysis (GEPIA2) and Gene Expression Omnibus (GEO) databases. We also explored SPOCK2 protein expression via the Clinical Proteomic Tumor Analysis Consortium (CPTAC) and Human Pathology Atlas Project (HPA) databases. GEPIA2 and Kaplan-Meier (KM) Plotter were employed as online bioinformatics tools to study the prognostic correlation between SPOCK2 expression and LUAD. We observed SPOCK2 was related to tumor stage (TNM classification) in LUAD using The Cancer Genome Atlas (TCGA) data. Then, Gene Ontology (GO) and Kyoto Encyclopedia of Genes and Genomes (KEGG) pathway enrichment analyses were conducted to assess the potential role of SPOCK2 in LUAD. Furthermore, the correlation between SPOCK2 and TIICs in LUAD was investigated in TCGA and GEO databases via TIMER. We also used the KM Plotter to examine the prognostic role of SPOCK2 with enriched and decreased TIIC subgroups. Our findings may shed light on the mechanism and role of SPOCK2 in LUAD.

## Materials and Methods

### Data Collection

The datasets analyzed during the current study are available in the GEO^1^ (Barrett et al., 2013) and TCGA repositories^2^ (Tomczak et al., 2015). The RNA sequencing fragments per kilobase million (FPKM) data and the corresponding clinical information were downloaded from TCGA-LUAD database. There were 594 samples in LUAD-TCGA, including 535 tumor samples and 59 normal samples. There were in total 352 tumor samples with complete information on age, gender, survival time, tumor stage, and TNM classification that were analyzed by SPSS to evaluate correlations between SPOCK2 expression and different clinicopathological factors. The microarray data collected from GEO was normalized by BART (Amaral et al., 2018).

### Detecting Differential Expression of SPOCK2

GEPIA2 (Tang et al., 2017) and the data downloaded from GSE32863 were used to evaluate the mRNA expression of SPOCK2. In the module “Expression DIY” of GEPIA2, differential expression between LUAD and normal controls were performed with the option of matching TCGA normal and GTEx data and log2(TPM + 1) for log-scale. GSE32863 met the criteria of gene expression profiling of LUAD and their matched histologically normal adjacent lung tissue samples which were applied for analyzing the differential expression of SPOCK2 between LUAD and normal lung tissues.

The CPTAC (Chen et al., 2019) database was applied to evaluate the total protein expression of SPOCK2 in LUAD. Log2 Spectral count ratio values were normalized within each sample profile and then across samples. The HPA (Ponten et al., 2011) database contains immunohistochemistry (IHC) data from 44 different normal tissue types and 17 major cancer types.

### Analysis of Prognostic Potential

The GEPIA2 and KM Plotter (Gyorffy et al., 2013) bioinformatics tools were applied to evaluate the prognostic potential of SPOCK2 in LUAD. There are three options for “Group Cutoff” in GEPIA2: “Median,” “Quartile,” and “Custom.” We chose that stratifying patients according to the “Median” expression of SPOCK2 in GEPIA2. Then, by choosing the “auto select best cutoff” option in KM Plotter, all possible cut-off values between the lower and upper quartiles are computed, and the best performing threshold is used as a cutoff.

### Gene Enrichment Analysis Based on SPOCK2-Coexpressed Genes

SPOCK2-coexpressed genes were displayed as a heatmap by LinkedOmics (Vasaikar et al., 2018) based on TCGA data and the genes were analyzed with the GO (Thomas, 2017) and KEGG pathway enrichment (Kanehisa et al., 2017) tools by R clusterProfiler (Yu et al., 2012) to visualize the mechanism of SPOCK2 in LUAD.

### RegNetwork Analysis

RegNetwork (Liu et al., 2015) is an online platform collecting experimentally validated and predicted gene regulations. Combinatorial and synergic regulatory correlations among transcription factors (TFs), miRNAs, and genes can be queried and identified in this regulatory network repository. A TF-miRNA-gene regulatory network was constructed by Cytoscape (Shannon et al., 2003).

### TIMER Database Analysis

The TIMER database (Li et al., 2017) is a bioinformatic web tool that can be applied to perform a comprehensive analysis of TIICs. The “Gene” module can evaluate the relationship between SPOCK2 mRNA expression and TIIC infiltration level using TCGA data, including data on B cells, CD8 + T cells, CD4 + T cells, neutrophils, macrophages, and dendritic cells. TIMER was also applied to explore the relationship between SPOCK2 mRNA expression and TIIC gene marker sets (Danaher et al., 2017; Siemers et al., 2017). Furthermore, the “Estimation” module in TIMER was used to measure the proportion of 22 immune cell subtypes in each LUAD sample from GSE37745, based on CIBERSORT^3^ (Newman et al., 2015). GSE37745 contains 106 LUAD samples. They were measured for the proportion of 22 immune cell subtypes of each LUAD sample. And then, they were evaluated for the correlation between SPOCK2 expression and the proportion of immune cell subtypes. The sample size is big enough and suitable for the bioinformatic tool “TIMER” and “Cibersort” to perform the calculation. CIBERSORT is a web portal which could characterize cell composition of complex tissues from the input gene expression profiles. It was used to measure the proportion of 22 immune cell subtypes in each LUAD sample from GSE37745.

### Statistical Analysis

Correlation assessment were carried out using SPSS version 17.0 (SPSS Inc., Chicago, IL, United States). The associations between SPOCK2 expression and the clinicopathological parameters of the LUAD patients were analyzed using the chi-squared test. Bivariate correlations between study variables were assessed with the Spearman’s rank correlation coefficient. *P* < 0.05 was considered statistically significant. Low and high SPOCK2 expression groups for correlation assessment were established according to the 60%-low and 40%-high SPOCK2 mRNA expression value in selected LUAD-TCGA dataset. Statistical analysis of SPOCK2 expression and TIICs relative proportion in the GEO dataset (GSE32863 and GSE37745) were performed using the GraphPad Prism 7 software (GraphPad Software Inc., La Jolla, CA, United States). Differences between the two groups were calculated by unpaired t-tests (^∗^*P* < 0.05, ^∗∗^*P* < 0.01, and ^∗∗∗^*P* < 0.001).

## Results

### SPOCK2 Was Downregulated in LUAD

**FIGURE 1:**
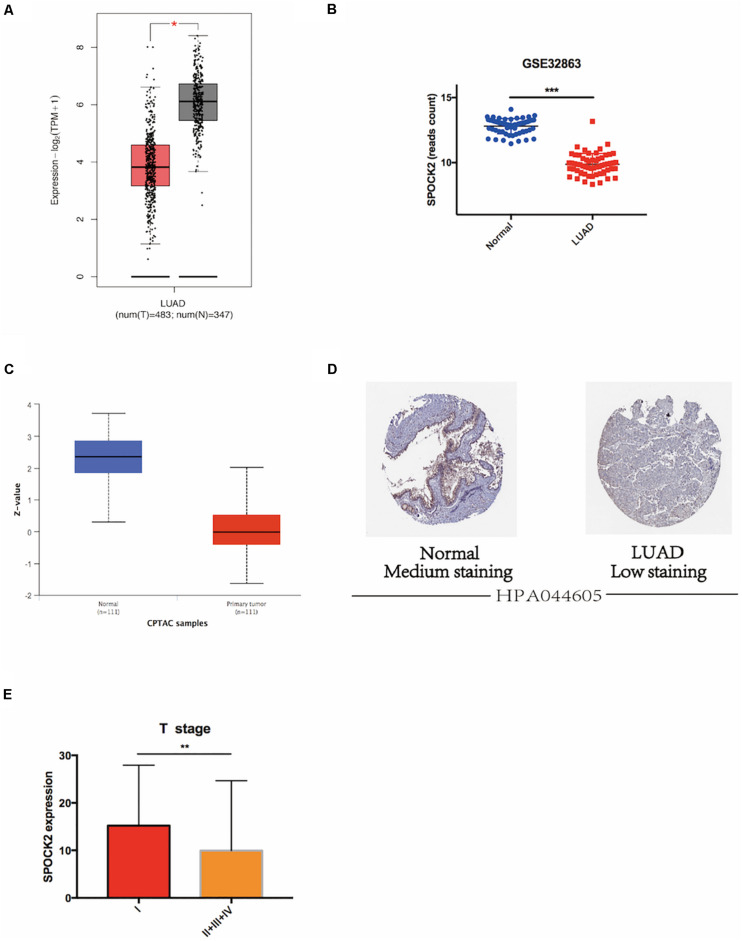
SPOCK2 expression in LUAD tissue at the mRNA level. **(A)** Differential SPOCK2 mRNA expression between LUAD and normal lung tissues in the GEPIA2 database. TPM: Transcripts Per Kilobase of exon model per Million mapped reads. **(B)** Differential SPOCK2 mRNA expression between LUAD and normal lung tissues in the GSE32863 dataset. **(C)** Differential SPOCK2 protein expression between LUAD and normal lung tissues in the CPTAC database. (Z-values: standard deviations from the LUAD median across samples). **(D)** Immunohistochemistry staining of SPOCK2 protein in LUAD and normal lung tissues in the HPA database. **(E)** Differential mRNA expression of SPOCK2 in LUAD with different T stages. FPKM: Fragments Per Kilobase of exon model per Million mapped fragments. *P < 0.05; **P < 0.01; ***P < 0.001.

We analyzed SPOCK2 mRNA expression in LUAD and normal lung tissue RNA sequencing data from the TCGA database via GEPIA2. The results revealed that the SPOCK2 mRNA expression level was lower in LUAD than in non-tumor lung tissue (*P* < 0.05, Figure 1A). This was validated in GSE32863 (*P* < 0.001, Figure 1B). We then investigated SPOCK2 protein expression in LUAD compared with that in normal lung tissues. The SPOCK2 protein expression level was lower in LUAD than in normal lung tissue in the CPTAC database (Figure 1C). Furthermore, IHC staining data from HPA database indicated that medium levels of SPOCK2 expression were present in normal lung tissues, while low levels of expression were observed in LUAD tissues (Figure 1D). Taken together, these results indicated that SPOCK2 was more highly expressed at the transcriptional and proteomic levels in normal lung tissues than in LUAD tissues.

### SPOCK2 Correlated With Clinical Parameters; High SPOCK2 Expression Correlated With Better Survival in LUAD Patients

**FIGURE 2:**
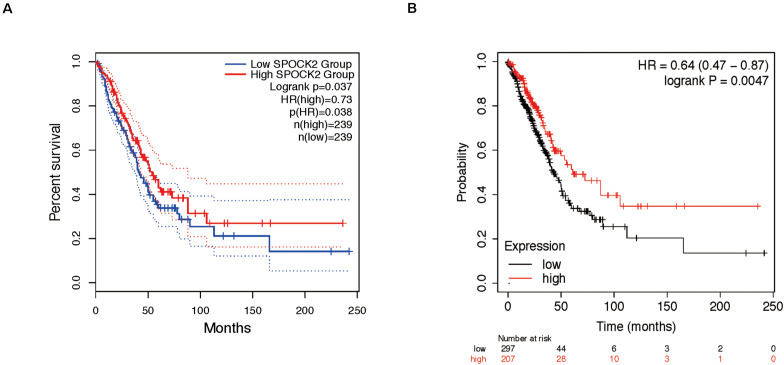
Prognostic role of SPOCK2 in LUAD. **(A)** Kaplan-Meier (KM) survival analysis of SPOCK2 in LUAD via GEPIA2. **(B)** KM survival analysis of SPOCK2 in LUAD via KM Plotter.

We analyzed the relationship between SPOCK2 and the clinicopathological characteristics of LUAD in the TCGA database. Differences in SPOCK2 mRNA expression were observed according to T stage classification, as shown in Figure 1E. Furthermore, SPOCK2 expression was observed to have a strong association with age (*P* = 0.001), clinical stage (*P* = 0.037), T classification (*P* < 0.001) and N classification (*P* = 0.012). However, SPOCK2 expression was not associated with sex (*P* = 0.111) or metastasis (*P* = 0.755) (Table 1). Similarly, spearman correlation analysis between SPOCK2 and clinicopathological characteristics revealed that the expression of SPOCK2 was significantly related to age (*P* = 0.001) and clinical stage (*P* = 0.015), T classification (*P* = 0) and N classification (*P* = 0.001) (Table 2). Then, the prognostic value of SPOCK2 in LUAD was analyzed by GEPIA2 database employing transcriptomic sequencing data (Figure 2A) and we found that a high SPOCK2 expression level correlated with better OS (HR (high) = 0.73, *P* (HR) = 0.038) in LUAD. Further, we used the KM Plotter to assess the prognostic value of SPOCK2 in LUAD and we found the similar result (HR = 0.64, logrank *P* = 0.0047) (Figure 2B). These results indicated a significant association between SPOCK2 expression and LUAD progression and prognosis.

**TABLE 1:** Correlation between SPOCK2 expression and clinicopathologic characteristics of LUAD.

Characteristics	SPOCK2		*P* value
	Low	High	
**Age (y)**			
≤60	88	31	0.001
>60	131	102	
**Gender**			
Male	101	73	0.111
Female	118	60	
**Clinical stage**			
I	103	81	0.037
II	57	28	
III	46	15	
IV	13	9	
**T classification**			
T1	43	59	<0.001
T2	142	60	
T3	21	9	
T4	13	5	
**N classification**			
N0	127	99	0.012
N1	50	22	
N2	41	12	
N3	1	0	
**Metastasis**			
No	206	124	0.755
Yes	13	9	

**TABLE 2:** Spearman correlation analysis between SPOCK2 and clinicopathological characteristics of LUAD.

Variables	SPOCK2 expression level	*p*-value
	**Spearman correlation**	
Age (y)	0.173	0.001
Gender	–0.085	0.111
Clinical stage	–0.129	0.015
T classification	–0.205	0
N classification	–0.174	0.001
Metastasis	0.017	0.765

### Functional Annotation and Pathway Enrichment of SPOCK2-Associated Genes and the SPOCK2 Regulatory Network

**FIGURE 3:**
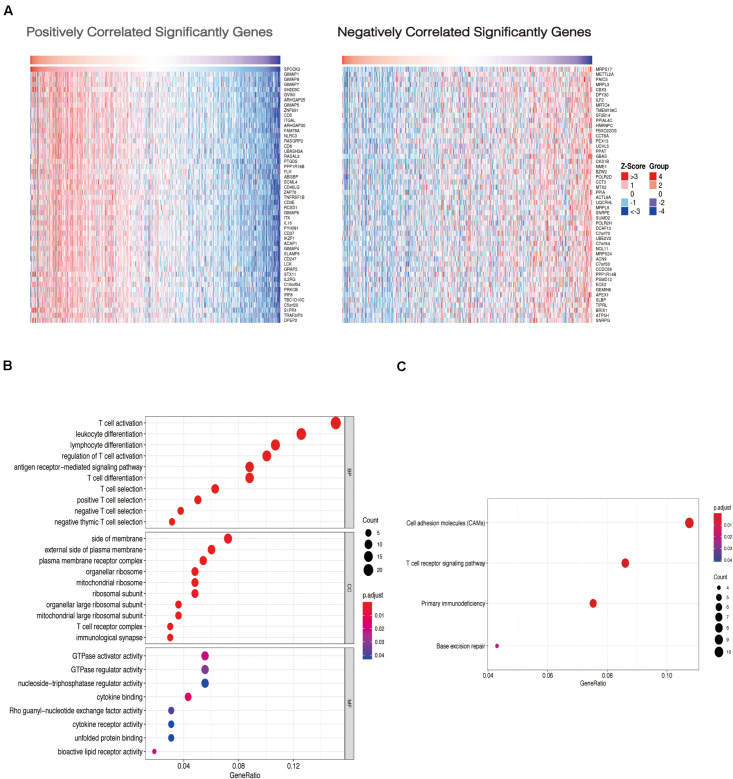
Gene enrichment analysis of SPOCK2 in LUAD datasets. **(A)** Heatmaps showing genes positively and negatively correlated with SPOCK2 in LUAD (top 50). **(B)** Enriched GO terms of SPOCK2-associated genes. **(C)** Enriched KEGG pathways of SPOCK2-associated genes.

**FIGURE 4:**
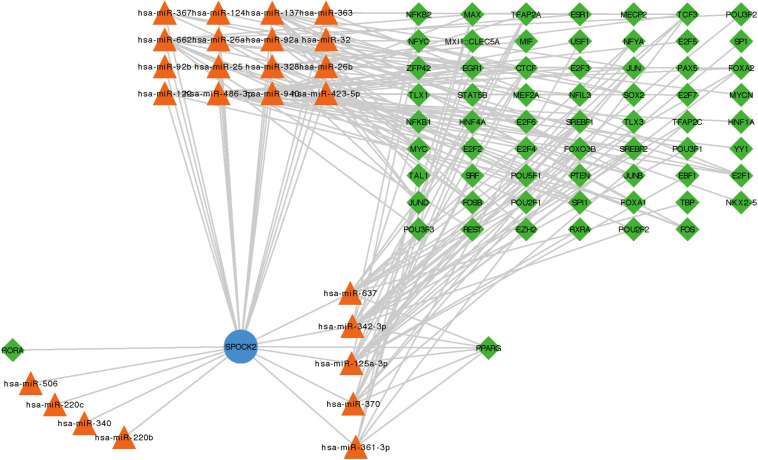
TF-SPOCK2-miRNA regulatory network.

To examine the co-expressed genes of SPOCK2, the LinkedOmics bioinformatics tool was employed to analyze mRNA sequencing data from LUAD patients in the TCGA database. The top 50 significant gene sets positively (left) and negatively (right) correlated with SPOCK2 were shown in the heatmap (Figure 3A). The result showed that SPOCK2 expression showed a strong correlation with GTPases of immunity-associated proteins (GIMAPs) family, including GIMAP1 (positive rank #1, Pearson correlation = 0.69, *p* = 5.45e–73), GIMAP8 (Pearson correlation = 0.69, *p* = 1.89e–72), and GIMAP7 (Pearson correlation = 0.68, *p* = 1.25e–70), which are regulators of lymphocyte survival and homeostasis (Schwefel et al., 2013). The result displayed the significant SPOCK2-correlated gene sets which help us explore the potential function of SPOCK2 by performing the enriched GO terms (Figure 3B) and KEGG pathways (Figure 3C) analysis. We discovered that these SPOCK2-associated genes were mainly enriched in lymphocyte activation, differentiation, and signaling, especially of T cells, with regard to biological process (BP) terms and KEGG pathway analysis results. Additionally, SPOCK2 was enriched in ribosome subunit and immunological synapse with regard to cellular component (CC) terms. In addition, we observed that SPOCK2 was involved in GTPase activity and cytokine binding with regard to molecular function (MF) terms. These results indicate the potential role of SPOCK2 to regulate tumor immunity in LUAD patients. In addition, RegNetwork database was employed to predict the TF and miRNA that could potentially bind to SPOCK2. We also utilized RegNetwork database to predict the potential binding of SPOCK2-associated TF and miRNA. Finally, TF (green)-miRNA (orange) co-regulatory interactions of SPOCK2 (blue) were constructed to study the potential SPOCK2 regulatory system (Figure 4).

### Relationship Between SPOCK2 Expression and TIICs

**FIGURE 5:**
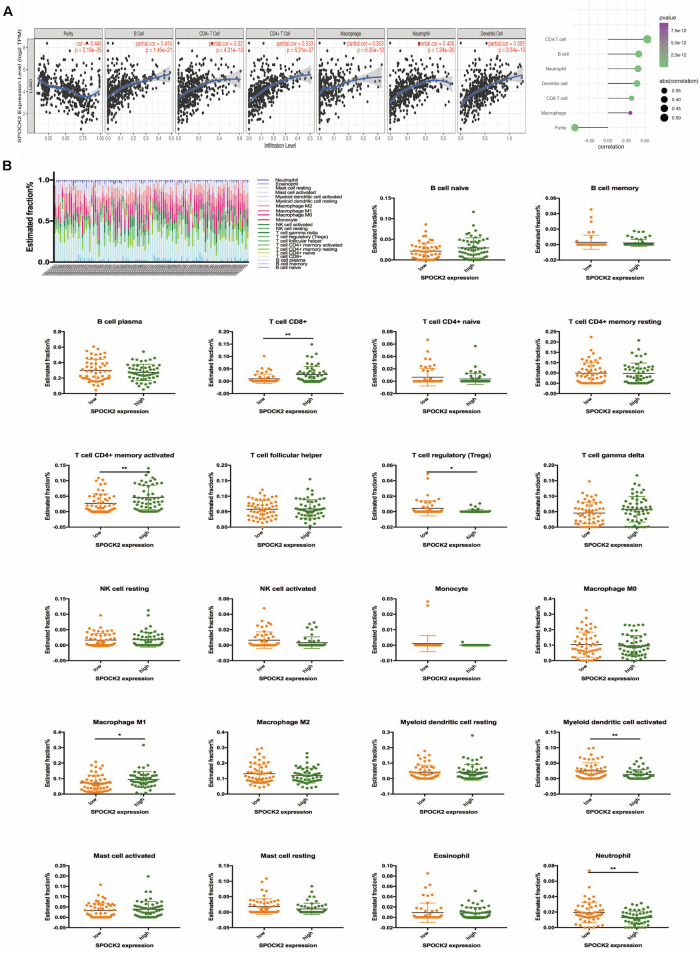
Correlation of SPOCK2 with TIICs in LUAD. **(A)** Correlation between SPOCK2 expression and immune infiltration in LUAD. **(B)** Based on 106 LUAD samples from the GSE37745 dataset, 22 kinds of TIICs are plotted according to the SPOCK2 expression level (horizontal lines indicate *p*-values <0.05; *P < 0.05; **P < 0.01).

Functional annotation and pathway enrichment indicated the potential role of SPOCK2 in regulating TIICs, which are vital in the prediction of the overall survival (OS) rate of LUAD. Therefore, we used TIMER to analyze possible associations between SPOCK2 expression and the TIIC infiltration level in LUAD. As shown in Figure 5A, SPOCK2 expression was positively associated with the levels of B cells (cor = 0.415, *P* = 1.45e–21), CD8 + T cells (cor = 0.32, *P* = 4.31e–13), CD4 + T cells (cor = 0.533, *P* = 6.31e–37), macrophages (cor = 0.303, *p* = 9.05e–12), neutrophils (cor = 0.406, *P* = 1.24e–20), and dendritic cells (cor = 0.392, *P* = 2.34e–19). These results indicated that SPOCK2 was important in regulating immune infiltration in LUAD. In addition, we observed via the TIMER database that SPOCK2 expression was strongly associated with the immunomarkers of TIICs and that these correlations remained unchanged after tumor purity correction (Table 3).

**TABLE 3:** Correlation analysis via TIMER between SPOCK2 and related genes and markers of immune cells in LUAD.

Description	Markers	SPOCK2			
		None		Purity	
		rho	*P*	rho	*P*
Neutrophils	CD11b (ITGAM)	0.4352	0	0.3271	9.37E–14
	CD66b (CEACAM8)	0.2256	2.28E–07	0.2352	1.24E–07
	CCR7	0.6467	0	0.5482	5.10E–40
Dendritic cell	HLA-DPB1	0.4911	1.28E–32	0.3837	9.80E-19
	HLA-DQB1	0.3762	9.24E–19	0.2609	4.08E–09
	HLA-DRA	0.4022	0	0.2682	1.43E–09
	HLA-DPA1	0.4381	0	0.3308	4.70E–14
	BDCA-1 (CD1C)	0.2986	4.56E–12	0.2931	3.06E–11
	BDCA-4 (NRP1)	0.1081	0.01415952	0.1066	1.78E–02
	CD11c (ITGAX)	0.5695	1.33E–45	0.4661	5.80E–28
T cell (general)	CD3D	0.5444	4.49E–41	0.5378	2.23E–38
	CD3E	0.6732	3.03E–69	0.5743	1.31E–44
	CD2	0.6289	4.76E–58	0.5158	7.24E–35
CD8 + T cell	CD8A	0.54	0	0.429	1.72E–23
	CD8B	0.4523	0	0.3562	3.40E–16
B cell	CD19	0.501	4.48E–34	0.3767	4.58E–18
	CD79A	0.4372	0	0.3064	3.57E–12
Monocyte	CD86	0.4417	0	0.2953	2.24E–11
	CD115(CSF1R)	0.4622	1.27E–28	0.3423	5.32E–15
TAM	CCL2	0.2743	2.86E–10	0.1383	0.00208767
	CD68	0.476	0	0.3782	3.30E–18
	IL10	0.4422	4.60E–26	0.3116	1.45E–12
M1 Macrophage	INOS(NOS2)	0.32	9.96E–14	0.2718	8.43E–10
	IRF5	0.3814	0	0.2771	3.84E–10
	COX2(PTGS2)	–0.091	0.03879957	–0.112	0.01285215
M2 Macrophage	CD163	0.4739	0	0.3627	8.95E–17
	VSIG4	0.3584	3.66E–17	0.2455	3.36E–08
	MS4A4A	0.4337	0	0.303	6.33E–12
Natural killer cell	KIR2DL1	0.2703	4.51E–10	0.2764	4.14E–10
	KIR2DL3	0.3057	1.34E–12	0.3102	1.77E–12
	KIR2DL4	0.1875	1.85E–05	0.1895	2.23E–05
	KIR3DL1	0.3089	7.61E–13	0.3204	2.95E–13
	KIR3DL2	0.3025	2.35E–12	0.286	9.35E–11
	KIR3DL3	0.1203	0.00625025	0.1169	0.00931353
	KIR2DS4	0.2839	5.29E–11	0.284	1.28E–10
Th1	TBX21	0.6263	0	0.5328	1.68E–37
	STAT1	0.3798	0	0.2845	1.23E–10
	STAT4	0.4651	5.27E–29	0.3404	7.70E–15
	TNF-α (TNF)	0.3625	9.90E–18	0.357	2.81E–16
	IFN-γ (IFNG)	0.3728	2.02E–18	0.368	2.76E–17
	CXCR3?	0.5581	0	0.4528	2.72E–26
Th2	CCR3	0.1995	5.08E–06	0.0985	0.02877601
	CCR4	0.5762	7.05E–47	0.4907	3.13E–31
	CCR7	0.6467	0	0.5482	5.10E–40
	CCR8	0.5087	3.05E–35	0.401	1.82E–20
	CD30 (TNFRSF8)	0.6407	0	0.5395	1.42E–38
	STAT6	0.256	4.26E–09	0.3006	9.34E–12
	STAT5A	0.6337	3.50E–59	0.5536	6.25E–41
Tfh	BCL6	0.1487	0.00071878	0.163	0.00027788
	IFNG	0.3728	2.02E–18	0.2533	1.18E–08
	TNF	0.3625	9.90E–18	0.2192	8.91E–07
	IL21	0.3038	1.86E–12	0.246	3.15E–08
Th17	STAT3	0.1568	0.00035389	0.1935	1.52E–05
	CCR6	0.5415	0	0.4353	3.29E–24
	IL17A	0.25	8.81E–09	0.1856	3.38E–05
Treg	FOXP3	0.5444	0	0.4288	1.83E–23
	CCR8	0.5087	3.05E–35	0.401	1.82E–20
	STAT5B	0.452	2.70E–27	0.4703	1.72E–28
	CD25 (IL2RA)	0.4289	1.83E–24	0.3019	7.61E–12
	TGFB1	0.3588	3.34E–17	0.261	4.05E–09

Further, we tried to examine whether the tumor immune microenvironment was different in LUAD patients with high SPOCK2 levels compared to those with low levels. The 106 LUAD samples from GSE37745 were divided into 2 groups based on median expression value of SPOCK2 expression value, with 53 samples in the high expression group and 53 samples in the low expression group. We used CIBERSORT by TIMER to examine the proportions of 22 types of immune cells by analyzing the gene expression profiles of these samples. We found that the proportions of several subtypes of T cell and activated lymphocyte subsets were significantly increased in the SPOCK2 high expression group compared with those in the SPOCK2 low expression group, including CD8 + T cells, activated memory CD4 + T cells, regulatory T cells, M1 macrophages, activated myeloid dendritic cells, and neutrophils (Figure 5B). Together, these results indicated that SPOCK2 plays a significant role in regulating TIICs in LUAD.

### Prognostic Analysis of SPOCK2 Expression in LUAD Based on TIICs Subsets

**FIGURE 6:**
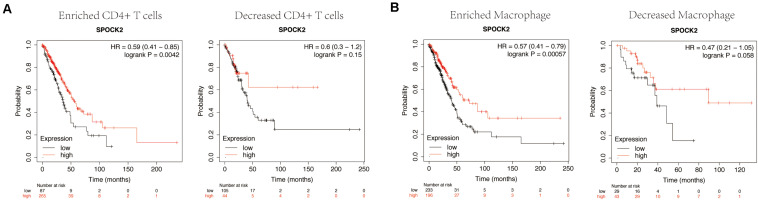
Comparison of Kaplan-Meier survival curves of SPOCK2 in LUAD based on enriched or decreased CD4 + T cells **(A)** and macrophage **(B)** subgroups.

We confirmed that SPOCK2 expression was positively correlated with favorable prognosis and TIICs in LUAD. Therefore, we speculated that the expression of SPOCK2 affects prognosis partly due to TIIC infiltration. We conducted prognostic analysis based on the SPOCK2 expression in enriched or decreased immune cell subgroups via KM Plotter. We observed that high expression levels of SPOCK2 in enriched CD4 + T cell (HR = 0.59) and enriched macrophage (HR = 0.57) cohorts were associated with a better prognosis, while there was no significant association among decreased subgroups (Figures 6A,B). These results supported our prediction that a high SPOCK2 expression level in LUAD affected prognosis partly because of the TIIC infiltration level.

## Discussion

Due to the poor outcomes reported in the latest cancer statistics released in 2019, there is an urgent need to identify novel prognostic markers for LUAD (Siegel et al., 2019). Cancer growth and spread are not only dependent on the characteristics of tumor cells but also on the interactions with components of the tumor microenvironment, especially TIICs (Hanahan and Weinberg, 2011; Matsushita et al., 2012; Steven et al., 2016), which positively correlate with better survival in LUAD (Iglesia et al., 2016). SPOCK2 correlates with the development and progression of various human cancers (Ren et al., 2011, 2019; Liu et al., 2019; Lou et al., 2019). SPOCK2 mRNA and protein are present in the lungs and are involved in alveolar development (Hadchouel et al., 2020) and bronchopulmonary dysplasia susceptibility (Hadchouel et al., 2011). However, the prognostic role of SPOCK2 in LUAD and the mechanism of the relationship between SPOCK2 and TIICs are still largely unknown.

In this study, we attempted to systematically explore the expression, prognostic value, correlation with TIICs, and potential mechanism of SPOCK2 in LUAD. In recent studies, SPOCK2 has been shown to be highly expressed in ovarian cancer (Lou et al., 2019) and lowly expressed in prostate cancer (Liu et al., 2019). Its expression increases during lung development (Hadchouel et al., 2020). We observed a significant decrease in SPOCK2 expression in LUAD compared with that in normal lung tissue at the mRNA level in the GEPIA2 and GEO databases (dataset GSE32863). We also found that SPOCK2 was downregulated in LUAD at the protein level in the CPTAC and HPA databases, indicating that SPOCK2 might be closely related to the occurrence and development of LUAD. To further study the potential role of SPOCK2 in lung cancer, we downloaded datasets from the TCGA database. We identified differential SPOCK2 expression in LUAD with T classification and SPOCK2 was negatively correlated with several clinical features including the pathological stage, tumor status, and lymph node status in LUAD patients. We then performed prognostic analysis using GEPIA2 and KM Plotter, and the results revealed that a high level of SPOCK2 expression was associated with better OS. SPOCK2 was previously reported to inhibit cancer cell invasion and migration in prostate cancer (Liu et al., 2019). Our findings indicated that SPOCK2 could constitute a promising prognostic biomarker in LUAD. However, the biological involvement of SPOCK2 in LUAD still needs to be explored.

To elucidate the molecular mechanisms underlying the role of SPOCK2 in LUAD, we explored the function of SPOCK2 and its coexpressed genes using LinkedOmics via GO and KEGG analysis with clusterProfiler. Most of the GO and KEGG categories were enriched in regulating lymphocytes, especially T cells based on LinkedOmics. TFs (Lambert et al., 2018) and miRNAs (Hayes et al., 2014) are central regulators of genes, functioning at the transcriptional and posttranscriptional levels, respectively (Le et al., 2015). We constructed a TF-SPOCK2-miRNA regulatory network using Cytoscape based on the RegNetwork database that may be greatly valuable for studying SPOCK2 regulatory systems because of its integration of prior knowledge.

SPOCK2 was reported to enhance anti-viral ability by inhibiting the cellular attachment and entry of the influenza virus (Ahn et al., 2019). In addition, the expression of SPOCK2 can be induced by interferon (IFN), which plays a vital role in immune responses in lung cancer. We speculated that viral infection-induced SPOCK2 expression may also be the result of immune system activation (Galani et al., 2017; Ahn et al., 2019). Therefore, we examined the correlation of SPOCK2 expression with the tumor immune system in LUAD.

We found that SPOCK2 was positively associated with the TIIC infiltration level among TCGA data, using TIMER. Moreover, the association between SPOCK2 expression and the marker genes of TIICs validated the role of SPOCK2 in LUAD tumor immunity.

TIIC subpopulations are different among cancer patients. For example, M1 macrophages and activated TIICs correlate with a relatively better prognosis (Pan et al., 2020). Likewise, in our study, significant correlations were found between SPOCK2 expression and several markers of TIICs in LUAD, which indicated that SPOCK2 plays a significant role in regulating the tumor immune microenvironment. Furthermore, we observed that the proportions of CD8 + T cells, activated memory CD4 + T cells, regulatory T cells, M1 macrophages, activated myeloid dendritic cells, and neutrophils increased in the SPOCK2 high expression group compared with those in the SPOCK2 low expression group in the GSE37745 dataset. Together, these results suggested that SPOCK2 played a vital role in regulating TIICs in LUAD.

Prognostic analysis of SPOCK2 expression levels based on immune cells in LUAD was performed using the KM Plotter. We observed that a high SPOCK2 expression level in the enriched CD4 + T cells and macrophage subgroups in LUAD was associated with a favorable prognosis. T cells and macrophages are closely associated with clinical outcome in LUAD (Iglesia et al., 2016) and our analysis results suggested that a high level of SPOCK2 expression in LUAD may affect the prognosis of LUAD cancer patients partly due to TIICs, indicating that SPOCK2 may have potential applications in immunotherapy.

To the best of our knowledge, the present study confirmed for the first time that SPOCK2 greatly affects LUAD prognosis. We found that a high expression level of SPOCK2 favored better survival in LUAD and correlated with TIICs. Therefore, SPOCK2 may affect prognosis partly due to its relationship with TIICs. Nonetheless, there were some limitations in our study. More LUAD patient samples are needed to confirm the prognostic value of SPOCK2, and the function of SPOCK2 in TIIC regulation in cancers as well as its influence on the response to immunotherapy should be verified in future clinical trials. In conclusion, SPOCK2 may be a useful biomarker and therapeutic target for LUAD prognosis and treatment, respectively.

## Data Availability Statement

The datasets GSE32863, GSE37745 and TCGA-LUAD for this study can be found in the GEO (http://www.ncbi.nlm.nih.gov/geo/) and TCGA (http://cancergenome.nih.gov) repositories.

## Author Contributions

QL: conceptualization. JZ: data curation, writing-original draft preparation. MC: methodology. JG, RZ, and TD: software. All authors contributed to the article and approved the submitted version.

## Conflict of Interest

The authors declare that the research was conducted in the absence of any commercial or financial relationships that could be construed as a potential conflict of interest.

## Footnotes

1 http://www.ncbi.nlm.nih.gov/geo/

2 http://cancergenome.nih.gov

3 http://cibersort.stanford.edu/
